# Supplementary material for: Application of the E-Nose as a Non-Destructive Technique in the Early Detection of Monilinia laxa on Plum (Prunus domestica L.)
Source: Sensors (Basel). 2025 Dec 13;25(24):7576. doi: 10.3390/s25247576 (PMC12737047; doi:10.3390/s25247576)
Supplement: Supplementary file 1 [file sensors-25-07576-s001.zip › Figure 1S.pdf]

Figure S1

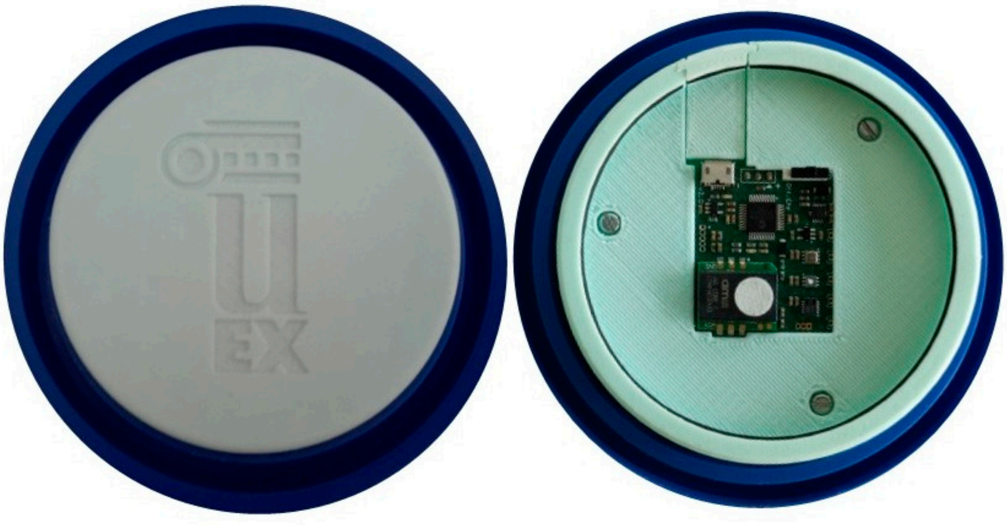

Figure S1. Components of the E-nose: on the left, the protective casing; on the right, the internal part where the electronic sensors are located.
