# Supplementary material for: Application of the E-Nose as a Non-Destructive Technique in the Early Detection of Monilinia laxa on Plum (Prunus domestica L.)
Source: Sensors (Basel). 2025 Dec 13;25(24):7576. doi: 10.3390/s25247576 (PMC12737047; doi:10.3390/s25247576)
Supplement: Supplementary file 1 [file sensors-25-07576-s001.zip › sensors-3982423-supplementary.pdf]

Table S1. Relative concentration (%) of volatile compounds identified in plum.

	Control (day 0)	No inoculated		*Monilinia laxa*		P values	
	C_C	C_1S	C_2S	M_1S	M_2S		
Alcohol							
3-methylbut-3-en-1-ol	0.00 ± 0.00	0.03 ± 0.03	0.04 ± 0.01	0.04 ± 0.01	0.05 ± 0.05		+++
2-methylbutan-1-ol	0.31 ± 0.12	0.34 ± 0.09	0.06 ± 0.04	0.10 ± 0.00	2.07 ± 0.61	+++	++
1-pentanol	0.00 ± 0.00	0.00 ± 0.00	0.00 ± 0.00	0.06 ± 0.08	0.27 ± 0.09		+++
3-hexenol	0.00 ± 0.00	0.00 ± 0.00	0.00 ± 0.00	0.20 ± 0.05	0.19 ± 0.08		
1-hexanol	0.10 ± 0.00	0.10 ± 0.03	0.34 ± 0.03	1.00 ± 0.12	1.77 ± 0.31		+++
Ethyl-1-hexanol	0.03 ± 0.01	0.04 ± 0.04	0.00 ± 0.00	0.01 ± 0.01	0.00 ± 0.00		
Phenylethyl alcohol	0.00 ± 0.00	0.00 ± 0.00	0.00 ± 0.00	0.00 ± 0.00	0.04 ± 0.05		
Ester							
Methyl acetate	0.00 ± 0.00	0.00 ± 0.00	0.00 ± 0.00	0.02 ± 0.03	0.12 ± 0.01	+	+++
Ethyl acetate	0.67 ± 0.34	1.26 ± 0.42	0.04 ± 0.06	0.68 ± 0.06	1.49 ± 0.25		++
n-propyl acetate	0.00 ± 0.00	0.00 ± 0.00	0.00 ± 0.00	0.06 ± 0.02	0.08 ± 0.09		+++
Methyl butanoate	0.00 ± 0.00	0.00 ± 0.00	0.00 ± 0.00	0.06 ± 0.01	0.35 ± 0.10	+++	
Isobutyl acetate	0.00 ± 0.00	0.03 ± 0.03	0.04 ± 0.01	0.04 ± 0.01	0.05 ± 0.05		+++
Ethyl butanoate	0.00 ± 0.00	0.06 ± 0.07	0.00 ± 0.00	0.12 ± 0.01	0.22 ± 0.02		
Butyl acetate	0.54 ± 0.09	2.89 ± 3.97	0.01 ± 0.00	0.96 ± 0.14	5.88 ± 1.71		+
Ethyl 2-methylbutanoate	1.87 ± 0.34	5.66 ± 4.72	5.78 ± 3.82	11.25 ± 0.34	3.76 ± 0.14	++	++
3-methylbutyl acetate	0.00 ± 0.00	0.09 ± 0.12	0.00 ± 0.00	0.00 ± 0.00	0.28 ± 0.10	+	+++
2-methylbutyl acetate	0.00 ± 0.00	0.00 ± 0.00	0.00 ± 0.00	0.05 ± 0.05	0.20 ± 0.01		
Propyl butanoate	0.00 ± 0.00	0.00 ± 0.00	0.00 ± 0.00	0.03 ± 0.03	0.00 ± 0.00		+++
Ethyl pentanoate	0.15 ± 0.03	0.13 ± 0.17	0.00 ± 0.00	0.20 ± 0.02	0.32 ± 0.01		
Butyl propanoate	0.00 ± 0.00	0.08 ± 0.11	0.00 ± 0.00	0.00 ± 0.00	0.13 ± 0.04		
Pentyl acetate	2.97 ± 0.31	2.14 ± 0.30	1.25 ± 0.69	2.07 ± 0.33	0.23 ± 0.08	---	-
Methyl hexanoate	0.00 ± 0.00	0.09 ± 0.11	0.12 ± 0.16	0.31 ± 0.03	0.09 ± 0.01		+++
Butyl 2-methylpropanoate	0.00 ± 0.00	0.00 ± 0.00	0.00 ± 0.00	0.41 ± 0.11	0.88 ± 0.19		
2-methylpropyl butanoate	0.17 ± 0.06	0.15 ± 0.01	0.10 ± 0.05	0.17 ± 0.00	0.02 ± 0.03	---	
2-methylbutyl propanoate	1.26 ± 0.15	0.49 ± 0.18	0.29 ± 0.27	0.27 ± 0.02	0.40 ± 0.07	---	

	Control (day 0)	No inoculated		*Monilinia laxa*		P values	
	C_C	C_1S	C_2S	M_1S	M_2S		
Butyl butanoate	0.07 ± 0.08	0.01 ± 0.00	0.00 ± 0.00	0.00 ± 0.00	0.00 ± 0.00		---
Ethyl hexanoate	4.02 ± 0.28	36.15 ± 1.44	2.60 ± 3.65	28.94 ± 2.41	25.12 ± 4.08	+++	+++
(Z)-3-hexenyl acetate	40.63 ± 2.86	3.62 ± 5.09	37.30 ± 20.11	3.74 ± 0.25	13.55 ± 3.53	---	--
Hexyl acetate	0.10 ± 0.01	0.23 ± 0.33	0.13 ± 0.10	1.00 ± 0.18	0.39 ± 0.44		++
Butyl 2-methylbutyrate	0.26 ± 0.03	0.40 ± 0.04	4.79 ± 1.95	6.64 ± 1.02	2.14 ± 0.38	++	
3-methybutyl butanoate	4.90 ± 0.05	4.41 ± 0.89	1.29 ± 0.72	1.42 ± 0.32	2.16 ± 0.42	---	
Isobutyl pentanoate	0.08 ± 0.01	0.02 ± 0.02	0.00 ± 0.00	0.00 ± 0.00	0.00 ± 0.00	---	
3-Methylbutyl isobutyrate	0.00 ± 0.00	0.00 ± 0.00	0.00 ± 0.00	0.00 ± 0.00	0.00 ± 0.00	---	---
2-methylbutyl butanoate	0.24 ± 0.01	0.07 ± 0.08	0.00 ± 0.00	0.11 ± 0.01	0.20 ± 0.02	---	+
4-pentenyl butyrate	3.11 ± 0.14	1.37 ± 0.62	1.10 ± 0.73	0.62 ± 0.11	0.55 ± 0.14	---	---
Pentyl pentanoate	0.00 ± 0.00	0.04 ± 0.04	0.11 ± 0.07	0.00 ± 0.00	0.00 ± 0.00	++	
Ethyl heptanoate	3.78 ± 0.19	2.81 ± 0.52	2.63 ± 1.08	2.18 ± 0.06	1.63 ± 0.44	-	---
3-methylbut-2-enyl butanoate	0.00 ± 0.00	0.00 ± 0.00	0.00 ± 0.00	0.00 ± 0.00	0.13 ± 0.04	+++	
Hexyl propanoate	0.38 ± 0.21	0.48 ± 0.16	0.53 ± 0.32	0.20 ± 0.09	0.37 ± 0.01		
Pentyl 2-methylbutyrate	0.12 ± 0.02	0.05 ± 0.07	0.26 ± 0.01	0.27 ± 0.05	0.04 ± 0.06		
Hexyl 2-methylpropanoate	0.14 ± 0.02	0.08 ± 0.11	0.00 ± 0.00	0.00 ± 0.00	0.00 ± 0.00	-	
Isobutyl hexanoate	0.00 ± 0.00	0.00 ± 0.00	0.03 ± 0.04	0.00 ± 0.00	0.00 ± 0.00		
2-methylbutyl pentanoate	0.79 ± 0.06	0.54 ± 0.28	0.86 ± 0.30	0.54 ± 0.07	0.65 ± 0.05		
(E)-3-hexenyl butanoate	0.05 ± 0.06	0.02 ± 0.03	0.00 ± 0.00	0.00 ± 0.00	0.00 ± 0.00		
Butyl hexanoate	0.71 ± 0.20	0.22 ± 0.13	0.24 ± 0.30	0.43 ± 0.11	0.13 ± 0.03	--	
Butyl (Z)-3-hexenoate	26.58 ± 1.83	30.50 ± 8.03	26.04 ± 28.30	30.62 ± 0.97	28.86 ± 0.59		+++
(3Z)-3-Hexenyl 2-methylbutanoate	0.23 ± 0.05	0.47 ± 0.34	0.11 ± 0.03	0.65 ± 0.06	1.25 ± 0.15		
Hexyl 2-methylbutanoate	0.08 ± 0.02	0.00 ± 0.00	0.00 ± 0.00	0.00 ± 0.00	0.00 ± 0.00	---	
Isopentyl hexanoate	0.68 ± 0.15	0.61 ± 0.44	0.52 ± 0.12	0.25 ± 0.01	0.41 ± 0.03		--
2-methylbutyl hexanoate	0.01 ± 0.00	0.00 ± 0.00	0.00 ± 0.00	0.05 ± 0.06	0.15 ± 0.00		+
4-pentenyl hexanoate	2.02 ± 0.47	1.61 ± 1.07	2.13 ± 0.11	0.83 ± 0.02	0.73 ± 0.03		---
Pentyl hexanoate	0.00 ± 0.00	0.00 ± 0.00	0.14 ± 0.01	0.00 ± 0.00	0.00 ± 0.00	+++	
3-methyl-2-butenylhexanoate	0.00 ± 0.00	0.05 ± 0.07	0.24 ± 0.09	0.02 ± 0.03	0.00 ± 0.00		

	Control (day 0)	No inoculated		*Monilinia laxa*		P values	
	C_C	C_1S	C_2S	M_1S	M_2S		
Hexyl hexanoate	0.83 ± 0.38	1.31 ± 0.97	8.28 ± 5.34	2.25 ± 0.11	1.48 ± 0.10		
Aldehyde							
Hexanal	0.01 ± 0.00	0.03 ± 0.03	0.00 ± 0.00	0.00 ± 0.00	0.04 ± 0.04		+
Octanal	0.04 ± 0.05	0.05 ± 0.05	0.06 ± 0.04	0.00 ± 0.00	0.00 ± 0.00		--
Nonanal	0.15 ± 0.16	0.00 ± 0.00	0.00 ± 0.00	0.00 ± 0.00	0.02 ± 0.02		
Decanal	0.01 ± 0.00	0.08 ± 0.01	0.08 ± 0.10	0.07 ± 0.00	0.11 ± 0.03		
Hydrocarbons							
Ethylbenzene	0.06 ± 0.07	0.04 ± 0.05	0.00 ± 0.00	0.00 ± 0.00	0.00 ± 0.00		
p-xylene	0.46 ± 0.14	0.28 ± 0.03	0.00 ± 0.00	0.01 ± 0.00	0.01 ± 0.00	--	---
Ketone							
3-pentanone	0.66 ± 0.06	0.00 ± 0.00	0.62 ± 0.12	0.28 ± 0.40	0.57 ± 0.16	-	-
Carboxilic acid							
(E)-3-octenoic acid	0.21 ± 0.09	0.27 ± 0.00	0.58 ± 0.28	0.34 ± 0.00	0.16 ± 0.01		
